# The Clinical Impact of Using ^18^F-FDG-PET/CT in the Diagnosis of Suspected Vasculitis: The Effect of Dose and Timing of Glucocorticoid Treatment

**DOI:** 10.1155/2019/9157637

**Published:** 2019-08-29

**Authors:** Kirsi Taimen, Soile P. Salomäki, Ulla Hohenthal, Markku Mali, Sami Kajander, Marko Seppänen, Pirjo Nuutila, Antti Palomäki, Anne Roivainen, Laura Pirilä, Jukka Kemppainen

**Affiliations:** ^1^Center for Rheumatology and Clinical Immunology, Division of Medicine, Turku University Hospital, Turku, Finland; ^2^Department of Medicine, University of Turku, Turku, Finland; ^3^Department of Infectious Diseases, Division of Medicine, Turku University Hospital, Turku, Finland; ^4^Turku PET Center, Turku University Hospital and University of Turku, Turku, Finland; ^5^Department of Radiology, Turku University Hospital, Turku, Finland; ^6^Department of Clinical Physiology and Nuclear Medicine, Turku University Hospital, Turku, Finland

## Abstract

^18^F-Fluorodeoxyglucose positron-emission tomography (^18^F-FDG-PET) with computed tomography (CT) is effective for diagnosing large vessel vasculitis, but its usefulness in accurately diagnosing suspected, unselected vasculitis remains unknown. We evaluated the feasibility of ^18^F-FDG-PET/CT in real-life cohort of patients with suspicion of vasculitis. The effect of the dose and the timing of glucocorticoid (GC) medication on imaging findings were in special interest. 82 patients with suspected vasculitis were evaluated by whole-body ^18^F-FDG-PET/CT. GC treatment as prednisolone equivalent doses at the scanning moment and before imaging was evaluated. 38/82 patients were diagnosed with vasculitis. Twenty-one out of 38 patients had increased ^18^F-FDG accumulation in blood vessel walls indicating vasculitis in various sized vessels. Vasculitis patients with a positive vasculitis finding in ^18^F-FDG-PET/CT had a significantly shorter duration of GC use (median = 4.0 vs 7.0 days, *P*=0.034), and they used lower GC dose during the PET scan (median dose = 15.0 mg/day vs 40.0 mg/day, *p*=0.004) compared to ^18^F-FDG-PET/CT-negative patients. Vasculitis patients with a positive ^18^F-FDG-PET/CT result had significantly higher C-reactive protein (CRP) than patients with a negative ^18^F-FDG-PET/CT finding (mean value = 154.5 vs 90.4 mg/L, *p*=0.018). We found that ^18^F-FDG-PET/CT positivity was significantly associated with a lower dose and shorter duration of GC medication and higher CRP level in vasculitis patients. ^18^F-FDG-PET/CT revealed clinically significant information in over half of the patients and was effective in confirming the final diagnosis.

## 1. Introduction

The diagnosis of vasculitis is a challenge, especially when vasculitis affects vital organs, and the patient presents nonspecific symptoms [1]. Vasculitis requires prompt recognition and initiation of treatment even if the diagnosis is uncertain.

The diagnostic process is often laborious. A biopsy is considered as a gold standard for diagnosing vasculitis, but in many cases, the optimal biopsy location is unavailable. The combination of ^18^F-fluorodeoxyglucose-positron emission tomography (^18^F-FDG-PET) with computed tomography (CT) is a promising diagnostic tool in the workup for vasculitis [2–4].

The accuracy and usefulness of ^18^F-FDG-PET/CT in the diagnostic procedure of vasculitis are still under debate. ^18^F-FDG-PET/CT has showed good performance in detecting large-vessel vasculitis (LVV) [4–7]. European League Against Rheumatism (EULAR) recommendation for the use of imaging in LVV in clinical practice recommends an early imaging test with ultrasound or MRI as first choices. PET may be used alternatively especially considering its ability to identify other serious differential diagnostic conditions [8]. In 2018, nuclear medicine interest committees gave a joint procedural recommendation on ^18^F-FDG-PET/CTA (angiography) imaging advising in data acquisition and interpretation in LVV and polymyalgia rheumatica [9]. Less is known about how ^18^F-FDG-PET/CT performs in other types of vasculitis than LVV. There is some evidence that PET may be useful in detecting small-vessel vasculitis [10, 11]. The ongoing multinational Diagnostic and Classification Criteria for Vasculitis (DCVAS) study aims to validate diagnostic criteria and to improve classification criteria for primary systemic vasculitis [12].

With a strong suspicion of vasculitis, rapid initiation of treatment is necessary. Glucocorticoids (GCs) are the most important first-line immunosuppressive treatment of noninfectious vasculitidies [13, 14]. Unfortunately, the use of immunosuppressive medication probably deteriorates the diagnostic accuracy of ^18^F-FDG-PET [7]. GC may attenuate ^18^F-FDG-uptake as early as after three days, but more confirmation is needed, since this is clinically a crucial question [15–18].

Here, we evaluated the impact of using ^18^F-FDG-PET/CT for accurately diagnosing vasculitis in real-life cohort of patients. We had a special interest in observing the effect of GC treatment prior to the ^18^F-FDG-PET/CT scan. We evaluated also differential diagnostic findings in patients with vasculitis suspicion.

## 2. Materials and Methods

### 2.1. Patients and Study Design

Eighty-two patients with suspected vasculitis were evaluated by whole-body ^18^F-FDG-PET/CT. The enrolment was done prospectively among inpatients. All diagnostic procedures were done at Turku University Hospital, Turku, Finland, between May 2011 and June 2015. The hospital is a tertiary-care centre for a population of 470 000. The institutional ethical committee approved the study protocol. All patients gave a written informed consent, according to the Declaration of Helsinki. This study is part of the Positron Emission Tomography of Infection and Vasculitis (PETU) study, which is registered as a clinical trial (NCT01878721). The PETU study researched different branches of infectious and inflammatory diseases which are reported separately [19–22]. Our series of vasculitis patients are previously unpublished.

The inclusion criterion of this study was vasculitis suspicion. Vasculitis suspicion was raised by an experienced specialist based on the clinical symptoms and signs of the patient. Vasculitis was confirmed or excluded by a consensus-based decision made by the specialists, while taking notice of the medical history, results of clinical examination, extensive laboratory work, ^18^F-FDG-PET/CT result, other imaging modalities, response to GC therapy, and follow-up. A minimum of 6 months clinical follow-up was considered sufficient to establish the diagnosis.

Special attention was paid to examine features and GC use in patients with diagnosed vasculitis in relation to ^18^F-FDG-PET/CT results. The cumulative GC dose was calculated from patients with a history of continuous GC use. GC use was evaluated as prednisolone equivalent doses.

### 2.2. Evaluation of the Diagnoses

The final diagnosis was based on the clinical picture as well as on the imaging findings of different sizes of affected vessels and histology. Based on the diagnosis, we divided the vasculitis patients into the following groups: LVV, medium- and small-vessel vasculitis, and unspecified vasculitis or antineutrophilic cytoplasmic antibodies- (ANCA-) associated vasculitis (AAV). Due to a low number of patients, vasculitis patients with granulomatous polyangiitis (GPA), eosinophilic granulomatous polyangiitis (EGPA), and microscopic polyangiitis (MPA) were combined into a group called AAV. In this group, five out of six patients were ANCA-positive, and an ANCA-negative patient had a histological finding of vasculitis.

All patients were evaluated by using the clinical criteria for vasculitis by American College of Rheumatology (ACR) 1990 [23, 24]. We evaluated the ACR criteria for GCA, GPA, EGPA, MPA, and polyarteritis nodosa (PAN). Due to a limited number of patients, cases from GPA, EGPA, and MPA formed a single group.

### 2.3. ^18^F-FDG-PET/CT Imaging Protocol

A whole-body ^18^F-FDG-PET/CT scan (64-slice Discovery VCT, General Electric Medical Systems, Milwaukee, WI, USA) was performed in all patients. Patients fasted at least 10 hours before the study. The mean injected radioactive dose of ^18^F-FDG was 273 MBq (range = 197–390 MBq). After an average of 57 minutes (range = 44–79 minutes), a whole-body PET acquisition (3 min/bed position) was performed following low-dose CT (kV 120, Smart mA range 10–80). In some patients, this was followed by a diagnostic contrast-enhanced CT scan (kV 120, Smart mA range 100–440) during the arterial phase after an automated i.v. injection of contrast agent.

Blood glucose levels were <10 mmol·L^−1^ prior to injection of the tracer in all patients. PET images were reconstructed in 128 × 128 matrix size in full 3D mode using maximum-likelihood reconstruction with an ordered-subset expectation maximization algorithm (VUE Point, GE Healthcare).

**Figure 1:**
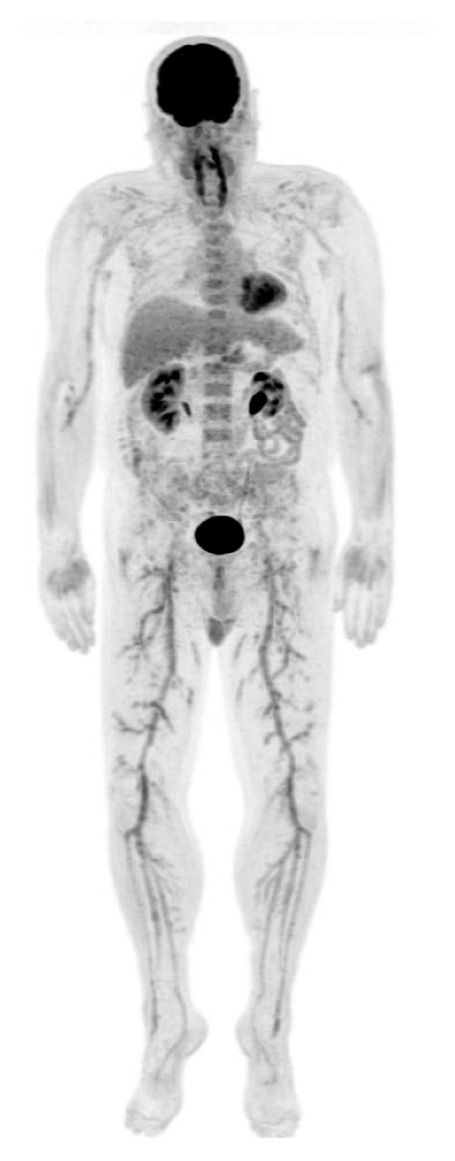
PET scan showing ^18^F-FDG-uptake in large- and medium- sized vessels. Maximum intensity projection (MIP) image of a whole-body PET-image of a 67-year-old male with high fever, mild headache, and a CRP value of 300 mg/l. After an extensive clinical workup, suspicion of vasculitis occurred, when there was no response to antibiotics. Whole-body CT showed no infection or malignant focus. Temporal arterial biopsy was equivocal. A PET/CT scan confirmed the vasculitis diagnosis by showing a tree-root-like ^18^F-FDG uptake pattern in large- and medium-sized arteries in the lower limbs. Physiological tracer uptake is noted in the brain, the neck muscles, the myocardium, the kidneys, and the bladder.

Visual analysis of the images was performed by an experienced nuclear medicine specialist, and the results were re-evaluated by the research team for a consensus-based diagnosis. All image analyses were done blinded with respect to patient's clinical details. ^18^F-FDG-PET/CT scans were considered positive, when a linear uptake pattern was found in the large arterial walls and/or its branches with an intensity similar or higher than the liver [25]. A positive finding for small- to medium-sized vasculitis was considered, when activity was higher than the vascular background activity and showed a tree-root-like uptake pattern [21] (Figure 1).

### 2.4. Statistical Analysis

Normally distributed continuous data were expressed as mean (standard deviation, SD), and for skewed distributions, data were expressed as median (interquartile range, IQR), unless stated otherwise. Categorical variables were described with absolute and relative (percentage) frequencies. An independent sample *t* test or Mann–Whitney *U* test was applied to determine the significance of differences for continuous variables as appropriate and a chi-squared or Fischer's exact test for categorical variables. All statistical analyses were calculated using SPSS Software Package (IBM SPSS Statistics Version 24). *P* values ≤0.05 were considered significant.

## 3. Results

### 3.1. Patients' Characteristics, Diagnosis, and ^18^F-FDG-PET/CT Findings

**Figure 2:**
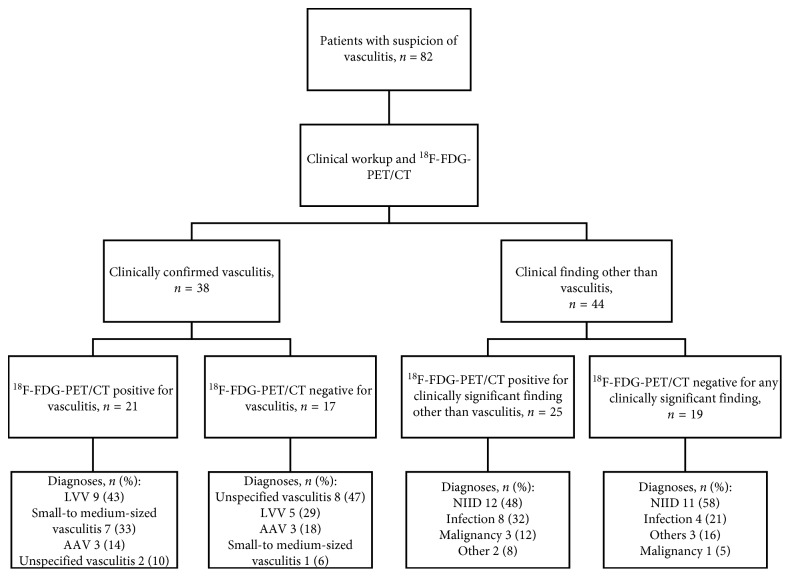
Diagram of the study design. 82 patients with a clinical suspicion of vasculitis referred for ^18^F-FDG-PET/CT were included. Diagnoses were confirmed by consensus-based decisions made by specialists after evaluation of a standard extensive workup, ^18^F-FDG-PET/CT scan, and a minimum of 6 months follow-up. Vasculitis patients with a negative ^18^F-FDG-PET/CT for vasculitis had other minor findings in PET/CT: mild infection (*n* = 2, 12%), pericarditis (*n* = 1, 6%), and pleuritis (*n* = 1, 6%). Among nonvasculitis patients, clinically significant ^18^F-FDG-PET/CT findings were as follows: NIID (*n* = 12), infection (*n* = 8), malignancy (*n* = 3), and miscellaneous (*n* = 2). LVV = large-vessel vasculitis. AAV = antineutrophil cytoplasmic antibody- (ANCA-) associated vasculitis. NIID = noninfectious inflammatory disease other than vasculitis.

A total of 82 patients with a clinical suspicion of vasculitis were referred for ^18^F-FDG-PET/CT and prospectively screened for this study (38 males and 44 females) (Figure 2). The mean age for patients was 62.7 years (age range = 19–89 years, SD = 16.0 years). An abnormal or clinically significant ^18^F-FDG-PET/CT finding was encountered in 46/82 patients (56%) (Table 1). A clinically significant ^18^F-FDG-PET/CT finding in different diagnostic subgroups is depicted in Table 1.

The vasculitis diagnosis was confirmed in 38 (46%) of the patients (Table 2). Most common cases of vasculitis were LVV (*n* = 14, 37%) and unspecified vasculitis (*n* = 10, 26%). Increased ^18^F-FDG accumulation in blood vessels suitable for vasculitis was detected in 21 of these 38 (55%) patients (Tables 2 and 3). ^18^F-FDG-PET/CT-positive patients fulfilled the ACR criteria for GCA significantly more often than ^18^F-FDG-PET/CT-negative patients (38% vs 8%, *p*=0.015). No accumulation of ^18^F-FDG in blood vessels was detected in 44 patients who did not fulfil the vasculitis diagnosis. Among patients without vasculitis diagnosis, the most common diagnostic groups were autoimmune diseases other than vasculitis (not including polymyalgia rheumatica, PM) (*n* = 18, 41%), infection (*n* = 12, 27%), PM (*n* = 5, 11%), and malignancy (*n* = 4, 9%) (Figure 2). In the PM group, one patient had ^18^F-FDG accumulation in the shoulder area relating to PM. One patient had a biopsy proven panniculitis which was clinically significant but not related to PM. Rest of the three patients did not have significant ^18^F-FDG-PET/CT findings.

### 3.2. Effect of Glucocorticoid Treatment on ^18^F-FDG-PET/CT Findings among the Vasculitis Patients

The duration and dose of GC treatment had a significant effect on the outcomes of the ^18^F-FDG-PET/CT scans. Out of 38 vasculitis patients, 9 patients (24%) had no GC treatment previously and 8 (21%) had used GC over 31 days.

**Figure 3:**
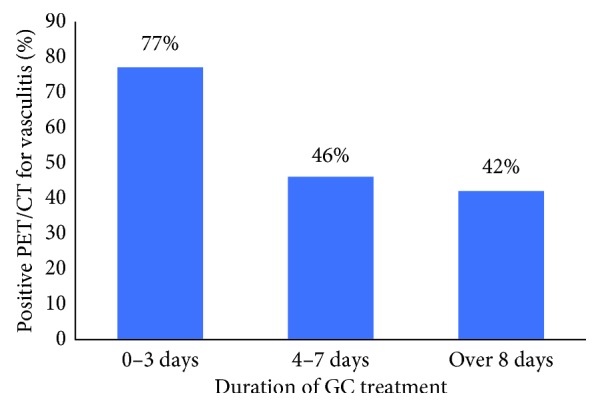
Positive ^18^F-FDG-PET/CT scans (%) for vasculitis and the duration of glucocorticoid (GC) treatment (days). In our study population, 21 of 38 vasculitis patients had positive ^18^F-FDG-PET/CT finding. In patients scanned within 3 days of GC treatment, 77% had vascular ^18^F-FDG uptake suitable for vasculitis in comparison to 42% after 8 days of treatment.

Vasculitis patients with positive ^18^F-FDG-PET/CT had significantly fewer days of GC use before imaging than patients with negative ^18^F-FDG-PET/CT (median = 4.0 (IQR 9) vs 7.0 (IQR 154) days, *p*=0.034) (Table 3). In patients scanned within 3 days of GC treatment, 77% had vascular ^18^F-FDG uptake suitable for vasculitis in comparison to 42% after one week of treatment (Figure 3). Among these 38 vasculitis patients, there was a significant association of ^18^F-FDG-PET/CT positivity with a lower GC dose on the scanning day with a median dose 15.0 (IQR 40.0) mg/day vs 40.0 (IQR 30.0) mg/day (*p*=0.004) (Table 3).

Patients with vasculitis used a higher GC dose during ^18^F-FDG-PET/CT scan than patients without vasculitis having a median prednisolone use of 30.0 (IQR 33.0) mg/day vs 0 (IQR 20.0) mg/day (*p*=0.001). Among vasculitis patients, 9 patients (24%) used no GC on the scanning day in comparison to the nonvasculitis group, where 24 patients (55%) used no GC on the scanning day.

### 3.3. Laboratory and Clinical Findings of the Patients

Among all 82 patients with suspicion of vasculitis, C-reactive protein (CRP) was elevated in 75 patients (91.5%), with a mean CRP value of 129.0 mg/L (SD = 89.5 mg/L). Vasculitis patients with a positive ^18^F-FDG-PET/CT scan had significantly higher CRP values than vasculitis patients with a negative ^18^F-FDG-PET/CT scan (mean CRP = 154.5 mg/L; SD 100.2 mg/L vs 90.4 mg/L; SD 55.6 mg/L, respectively; *p*=0.018) (Table 3). No difference was found in procalcitonin (PCT) values (data available from 62 patients) between vasculitis patients with positive or negative ^18^F-FDG-PET/CT findings (Table 3). There was no difference in CRP or PCT values between vasculitis and nonvasculitis patients (Table 2).

Forty-eight (out of 79) patients (60.8%) had a fever over 38°C. Other common clinical symptoms were haematuria (*n* = 38/75, 46.3%), myalgia (*n* = 36/79, 43.9%), hip pain (*n* = 24/76, 29.3%), bilateral shoulder pain (*n* = 18/78, 22%), a new headache (*n* = 14/78, 17.1%), and new neuropathy (*n* = 11/79, 13.3%). When comparing vasculitis with nonvasculitis patients, vasculitis patients had significantly more often a new headache (29% vs 7%, *p*=0.008).

## 4. Discussion

The spectrum of conditions causing vasculitis-like symptoms is wide. We found that in real-life cohort of patients, ^18^F-FDG-PET/CT was effective in confirming the final diagnosis among inpatients with vasculitis suspicion. ^18^F-FDG-PET/CT showed vasculitis in 26% of all patients and revealed clinically significant information in over half of the patients.

We found that among vasculitis patients, a shorter duration of prednisolone use is significantly associated with positive ^18^F-FDG-PET/CT vasculitis findings (Table 3). Vasculitis patients with positive ^18^F-FDG-PET/CT imaging had a median of 4 days of prednisolone treatment versus 7 days in the negative ^18^F-FDG-PET/CT group. This implicates that withholding diagnostic imaging for over one week during GC treatment increases the risk of a false-negative diagnosis. In the vasculitis group, a lower GC dose at the scanning moment was significantly associated with an ^18^F-FDG-PET/CT-based vasculitis diagnosis (Table 3).

In a previous study, good sensitivity, at 80%, and specificity, at 79%, have been reported for ^18^F-FDG-PET/CT in patients with GCA receiving GC less than 3 days [6]. In another study, Fuchs et al. reported that the sensitivity of ^18^F-FDG-PET/CT lowers from 99% to 53% in patients with GCA receiving an immunosuppressant [7]. A reduction of ^18^F-FDG accumulation under treatment has been reported in follow-up studies [16, 26]. A study by Imfeld et al. shows that prednisone treatment ≥10 days significantly reduced ^18^F-FDG-PET/CT sensitivity. The first effect of lowered sensitivity was seen as early as 3 days after treatment initiation in the abdominal aorta [17] and in supra-aortic vessels [15]. Surprisingly, a study by Clifford et al. [18] did not find a correlation. Clifford et al. explained that their study subject number was low (*n* = 28), patients had received treatment over long time (on an average of 11.9 days), and doses were similarly high among all patients.

In a real clinical setting, withholding the treatment initiation until imaging is often impossible, so the knowledge of the GC effect on ^18^F-FDG-PET diagnostic performance is important. Our study supports the data that GC treatment reduces the diagnostic power of ^18^F-FDG-PET/CT after one week. Thus, there is a need for fast ^18^F-FDG-PET/CT availability for suspected vasculitis patients. These patients represent often a diffuse clinical picture. Ultrasound, which nowadays is the recommended standard protocol in LVV, performs poorly in thoracic aorta area or in small, deep vessels without focal symptoms. In our material, ^18^F-FDG-PET/CT was useful also in other vasculitis than LVV and performs well in thoracic vessels. A lower GC dose during PET/CT scanning was associated with vasculitis findings in ^18^F-FDG-PET/CT, but our study cannot answer the question that, if lowering temporarily the GC dose helps avoid false-negative results. In few patients, ^18^F-FDG-PET/CT showed vascular uptake suitable for vasculitis even after long GC treatment. In our cohort, the duration of use and dosage of GC treatment varied in patients at the ^18^F-FDG-PET/CT imaging due to the study design.

We found a significant correlation between higher CRP value and ^18^F-FDG-PET/CT positivity in patients diagnosed with vasculitis. A high CRP value might reflect more active inflammation and less use of GC at the scanning moment. There are several studies testing the correlation of laboratory parameters and diagnostic performance of ^18^F-FDG-PET/CT in GCA, in fever of unknown origin (FUO), or in inflammation of unknown origin (IUO) [17, 27–29]. FUO and IUO are essential differential diagnostic challenges for vasculitis. Schönau et al. reported that an age over 50 years, a CRP level over 30 mg/L, and the absence of fever predicted the helpfulness of ^18^F-FDG-PET/CT [27] in FUO and IUO. Papathanasiou et al. noticed a significant positive association between maximal aortic ^18^F-FDG uptake and inflammatory markers [29].

Our study had limitations that should be considered. The study was done in a real clinical setting, and the inclusion criterion was vasculitis suspicion; therefore, the vasculitis patient group was heterogeneous. The vasculitis diagnosis was confirmed later, and the spectrum of different vasculitis was detected. Our study did not exclude patients who did not fulfil the ACR inclusion criteria. This might be a limitation when comparing the results to previous studies with more restricted inclusion criteria.

## 5. Conclusions

We found that in patients with confirmed vasculitis diagnosis, ^18^F-FDG-PET/CT positivity was significantly related to a lower dose and shorter duration of GC medication and a higher CRP level. In real-life circumstances, ^18^F-FDG-PET/CT revealed different types of vasculitidies as well as other clinically significant information in over half of the patients and had an impact in confirming the final diagnosis.

## Figures and Tables

**Table 1:** Final clinical diagnosis and significance of PET/CT by each diagnosis.

Category	Number of cases	Clinically significant PET/CT finding
Other autoimmune diseases	18	10/18
Adult-onset Still's disease	3	0/3
Sarcoidosis	2	1/2
Collagenosis	2	2/2
Pericarditis	2	1/2
Morbus Crohn/IBD	2	1/2
Myositis	2	2/2
SLE	2	1/2
Unspecified	2	1/2
Rheumatoid arthritis	1	1/1

Large vessel vasculitis	14	9/14
Giant cell arteritis	13	9
Takayasu arteritis	1	0

Infection	12	8/12
Infection NAS/FUO	3	2/3
Deep abscess	3	2/3
Septic arthritis	1	1/1
Septic spondylodiscitis	1	1/1
Pneumonia	1	1/1
Urinary tract infection	1	0/1
Cholecystitis	1	1/1
Tuberculosis	1	0/1

Unspecified vasculitis^*∗*^	10	2/10
Vasculitis NAS	8	2
Secondary vasculitis	2	0

Small- and medium-sized vasculitis (other than ANCA-associated vasculitis)	8	7/8

ANCA-associated vasculitis^*∗∗*^	6	3/6
EGPA	3	1/3
GPA	2	2/2
MPA	1	0/1

Polymyalgia rheumatica	5	2/5

Malignancy	4	3/4
Lymphoma	3	2/3
Lung cancer	1	1/1

Miscellaneous	4	1/4
Cardiac disease	2	0/2
Calciphylaxis	1	0/1
Leg ulcers	1	1/1

Unknown diagnosis	1	1/1

ANCA, antineutrophil cytoplasmic antibody; EGPA, eosinophilic granulomatosis with polyangiitis; GPA, granulomatosis with polyangiitis; MPA, microscopic polyangiitis; FUO, fever of unknown origin. ^*∗*^Vasculitis diagnosis confirmed by either imaging or biopsy. ^*∗∗*^5/6 patients were ANCA-positive. The ANCA-negative patient had biopsy confirmed diagnosis.

**Table 2:** Patients' characteristics based on vasculitis diagnosis.

	Vasculitis (*n* = 38)	No vasculitis (*n* = 44)	*P* value
Female sex, *n* (%)	23 (60.5)	21 (47.7)	0.246
Age, years, mean (SD)	66.3 (13.4)	59.5 (17.5)	0.056
CRP max, mg/l, mean (SD)	125.8 (88.3)	131.8 (91.4)	0.765
PCT max, *µ*g/l, mean (SD)	0.16 (0.16), *n* = 29	0.16 (0.18), *n* = 33	0.872
Prednisolone at scanning moment, mg, median (IQR)	30.0 [33]	1.0 [20]	0.001^*∗*^
Patients using prednisolone	29/38	20/44	
Prednisolone prior scanning, *d*, median (IQR)	6.0 [11]	0.0 [52]	0.135
Prednisolone cumulative dose, mg, median (IQR)	260.0 [1500]	1.00 [1706]	0.075
Fulfills ACR criteria for GCA, *n* (%)	10 (26.3)	3 (6.8)	0.016^*∗*^
Fulfills ACR criteria for EGPA, GPA, or MPA, *n* (%)	12 (31.6)	8 (18.2)	0.159
Fulfills ACR criteria for PAN, *n* (%)	5 (13.2)	2 (4.5)	0.164
Fever over 38°C, *n* = 79, *n* (%)	22 (57.9)	26 (63.4)	0.616

SD, standard deviation; CRP, C-reactive protein; PCT, procalcitonin; IQR, interquartile range; ACR, American College of Rheumatology; GCA, giant cell arteritis; EGPA, eosinophilic granulomatous polyangiitis; GPA, granulomatous polyangiitis; MPA, microscopic polyangiitis; PAN, polyarteritis nodosa. ^*∗*^Significant at *P* value <0.05.

**Table 3:** Characteristics of vasculitis patients.

	^18^F-FDG-PET/CT positive (*n* = 21)	^18^F-FDG-PET/CT negative (*n* = 17)	*P* value
Female sex, *n* (%)	14 (66.7)	9 (52.9)	0.389
Age, years, mean (SD)	68.0 (12.1)	64.2 (15.0)	0.390
CRP max, mg/l, mean (SD)	154.5 (100.2)	90.4 (55.6)	0.018^*∗*^
PCT max, *μ*g/l, mean (SD)	0.12 (0.09), *n* = 17	0.22 (0.02), *n* = 12	0.137
ANCA positive, *n* (%)	3 (14.3)	4 (23.5)	0.478
Prednisolone at scanning moment, mg, median [IQR]	15.0 [40.0]	40.0 [30.0]	0.004^*∗*^
Prednisolone prior scanning, *d*, median [IQR]	4.0 [9]	7.0 [154]	0.034^*∗*^
Prednisolone cumulative dose, mg, median [IQR]	120 [1120]	360 [1965]	0.096
Fever over 38°C	14 (66.7)	8 (47.1)	0.224

SD, standard deviation; CRP, C-reactive protein; PCT, procalcitonin; IQR, interquartile range; ANCA, antineutrophil cytoplasmic antibody. ^*∗*^Significant at *P* value <0.05.

## Data Availability

The data included in this study are available upon request from the corresponding author.

## References

[1] Weyand C. M., Goronzy J. J. (2014). Giant-cell arteritis and polymyalgia rheumatica. *New England Journal of Medicine*.

[2] Prieto-González S., Arguis P., Cid M. C. (2015). Imaging in systemic vasculitis. *Current Opinion in Rheumatology*.

[3] Puppo C., Massollo M., Paparo F. (2014). Giant cell arteritis: a systematic review of the qualitative and semiquantitative methods to assess vasculitis with ^18^F-fluorodeoxyglucose positron emission tomography. *BioMed Research International*.

[4] Besson F. L., Parienti J.-J., Bienvenu B. (2011). Diagnostic performance of ^18^F-fluorodeoxyglucose positron emission tomography in giant cell arteritis: a systematic review and meta-analysis. *European Journal of Nuclear Medicine and Molecular Imaging*.

[5] Soussan M., Nicolas P., Schramm C. (2015). Management of large-vessel vasculitis with FDG-PET. *Medicine*.

[6] Prieto-González S., Depetris M., García-Martínez A. (2014). Positron emission tomography assessment of large vessel inflammation in patients with newly diagnosed, biopsy-proven giant cell arteritis: a prospective, case-control study. *Annals of the Rheumatic Diseases*.

[7] Fuchs M., Briel M., Daikeler T. (2012). The impact of ^18^F-FDG PET on the management of patients with suspected large vessel vasculitis. *European Journal of Nuclear Medicine and Molecular Imaging*.

[8] Dejaco C., Ramiro S., Duftner C. (2018). EULAR recommendations for the use of imaging in large vessel vasculitis in clinical practice. *Annals of the Rheumatic Diseases*.

[9] Slart R. H. J. A., Writing Group, Reviewer Group (2018). FDG-PET/CT(A) imaging in large vessel vasculitis and polymyalgia rheumatica: joint procedural recommendation of the EANM, SNMMI, and the PET interest group (PIG), and endorsed by the ASNC. *European Journal of Nuclear Medicine and Molecular Imaging*.

[10] Soussan M., Abisror N., Abad S. (2014). FDG-PET/CT in patients with ANCA-associated vasculitis: case-series and literature review. *Autoimmunity Reviews*.

[11] Kemna M. J., Vandergheynst F., Vöö S. (2015). Positron emission tomography scanning in anti-neutrophil cytoplasmic antibodies-associated vasculitis. *Medicine*.

[12] Luqmani R. A., Suppiah R., Grayson P. C., Merkel P. A., Watts R. (2011). Nomenclature and classification of vasculitis—update on the ACR/EULAR diagnosis and classification of vasculitis study (DCVAS). *Clinical and Experimental Immunology*.

[13] Buttgereit F., Dejaco C., Matteson E. L., Dasgupta B. (2016). Polymyalgia rheumatica and giant cell arteritis. *JAMA*.

[14] Muratore F., Pazzola G., Soriano A. (2018). Unmet needs in the pathogenesis and treatment of vasculitides. *Clinical Reviews in Allergy and Immunology*.

[15] Nielsen B. D., Gormsen L. C., Hansen I. T., Keller K. K., Therkildsen P., Hauge E.-M. (2018). Three days of high-dose glucocorticoid treatment attenuates large-vessel ^18^F-FDG uptake in large-vessel giant cell arteritis but with a limited impact on diagnostic accuracy. *European Journal of Nuclear Medicine and Molecular Imaging*.

[16] Blockmans D., De Ceuninck L., Vanderschueren S., Knockaert D., Mortelmans L., Bobbaers H. (2006). Repetitive ^18^F-fluorodeoxyglucose positron emission tomography in giant cell arteritis: a prospective study of 35 patients. *Arthritis and Rheumatism*.

[17] Imfeld S., Rottenburger C., Schegk E. (2017). [^18^F]FDG positron emission tomography in patients presenting with suspicion of giant cell arteritis—lessons from a vasculitis clinic. *European Heart Journal-Cardiovascular Imaging*.

[18] Clifford A. H., Murphy E. M., Burrell S. C. (2017). Positron emission tomography/computerized tomography in newly diagnosed patients with giant cell arteritis who are taking glucocorticoids. *The Journal of Rheumatology*.

[19] Salomäki S. P., Saraste A., Kemppainen J. (2017). ^18^F-FDG positron emission tomography/computed tomography in infective endocarditis. *Journal of Nuclear Cardiology*.

[20] Salomaki S. P., Hohenthal U., Kemppainen J., Pirila L., Saraste A. (2014). Visualization of pericarditis by fluorodeoxyglucose PET. *European Heart Journal-Cardiovascular Imaging*.

[21] Salomäki S. P., Kemppainen J., Aho H. (2014). Widespread vascular inflammation in a patient with antineutrophil cytoplasmic antibody-associated vasculitis as detected by positron emission tomography. *European Journal of Nuclear Medicine and Molecular Imaging*.

[22] Salomäki S. P., Kemppainen J., Hohenthal U. (2017). Head-to-head comparison of ^68^Ga-citrate and ^18^F-FDG PET/CT for detection of infectious foci in patients with *Staphylococcus aureus* bacteraemia. *Contrast Media & Molecular Imaging*.

[23] Hunder G. G., Bloch D. A., Michel B. A. (1990). The American college of rheumatology 1990 criteria for the classification of giant cell arteritis. *Arthritis and Rheumatism*.

[24] Fries J. F., Hunder G. G., Bloch D. A. (1990). The American college of rheumatology 1990 criteria for the classification of vasculitis: summary. *Arthritis and Rheumatism*.

[25] Jamar F., Buscombe J., Chiti A. (2013). EANM/SNMMI guideline for ^18^F-FDG use in inflammation and infection. *Journal of Nuclear Medicine*.

[26] Martínez-Rodríguez I., Jiménez-Alonso M., Quirce R. (2018). ^18^F-FDG PET/CT in the follow-up of large-vessel vasculitis: a study of 37 consecutive patients. *Seminars in Arthritis and Rheumatism*.

[27] Schönau V., Vogel K., Englbrecht M. (2018). The value of ^18^F-FDG-PET/CT in identifying the cause of fever of unknown origin (FUO) and inflammation of unknown origin (IUO): data from a prospective study. *Annals of the Rheumatic Diseases*.

[28] Balink H., Veeger N. J. G. M., Bennink R. J. (2015). The predictive value of C-reactive protein and erythrocyte sedimentation rate for ^18^F-FDG PET/CT outcome in patients with fever and inflammation of unknown origin. *Nuclear Medicine Communications*.

[29] Papathanasiou N. D., Du Y., Menezes L. J. (2012). ^18^F-fludeoxyglucose PET/CT in the evaluation of large-vessel vasculitis: diagnostic performance and correlation with clinical and laboratory parameters. *The British Journal of Radiology*.

